# False Positive Multiparametric Magnetic Resonance Imaging Phenotypes in the Biopsy-naïve Prostate: Are They Distinct from Significant Cancer-associated Lesions? Lessons from PROMIS^☆^

**DOI:** 10.1016/j.eururo.2020.09.043

**Published:** 2021-01

**Authors:** Vasilis Stavrinides, Tom Syer, Yipeng Hu, Francesco Giganti, Alex Freeman, Solon Karapanagiotis, Simon R.J. Bott, Louise C. Brown, Nicholas Burns-Cox, Timothy J. Dudderidge, Ahmed El-Shater Bosaily, Elena Frangou, Maneesh Ghei, Alastair Henderson, Richard G. Hindley, Richard S. Kaplan, Robert Oldroyd, Chris Parker, Raj Persad, Derek J. Rosario, Iqbal S. Shergill, Lina M. Carmona Echeverria, Joseph M. Norris, Mathias Winkler, Dean Barratt, Alex Kirkham, Shonit Punwani, Hayley C. Whitaker, Hashim U. Ahmed, Mark Emberton, on behalf of the PROMIS group

**Affiliations:** a UCL Division of Surgery & Interventional Science, University College London, London, UK; b The Alan Turing Institute, London, UK; c Department of Urology, University College London Hospitals NHS Foundation Trust, London, UK; d Centre for Medical Imaging, University College London, London, UK; e Centre for Medical Image Computing, University College London, London, UK; f Wellcome EPSRC Centre for Interventional & Surgical Science (WEISS), University College London, London, UK; g Department of Medical Physics & Biomedical Engineering, University College London, London, UK; h Department of Radiology, University College London Hospitals NHS Foundation Trust, London, UK; i Department of Pathology, University College London Hospitals NHS Foundation Trust, London, UK; j Medical Research Council (MRC) Biostatistics Unit, University of Cambridge, Cambridge, UK; k Department of Urology, Frimley Health NHS Foundation Trust, London, UK; l Medical Research Council (MRC) Clinical Trials Unit, University College London, London, UK; m Department of Urology, Taunton & Somerset NHS Foundation Trust, Taunton, UK; n Department of Urology, University Hospital Southampton NHS Foundation Trust, Southampton, UK; o Department of Radiology, Royal Free London NHS Foundation Trust, London, UK; p Department of Urology, Whittington Health NHS Trust, London, UK; q Department of Urology, Maidstone & Tunbridge Wells NHS Trust, Tunbridge Wells, UK; r Department of Urology, Hampshire Hospitals NHS Foundation Trust, Hampshire, UK; s Public and patient representative, Nottingham, UK; t Department of Academic Urology, The Royal Marsden NHS Foundation Trust, Sutton, UK; u Department of Urology, North Bristol NHS Trust, Bristol, UK; v Department of Urology, Sheffield Teaching Hospitals NHS Foundation Trust, Sheffield, UK; w Department of Urology, Wrexham Maelor Hospital NHS Trust, Wrexham, UK; x Department of Urology, Imperial College Healthcare NHS Trust, London, UK; y Imperial Prostate, Division of Surgery, Department of Surgery & Cancer, Faculty of Medicine, Imperial College London, London, UK

**Keywords:** False positive lesions, Multiparametric magnetic resonance imaging, PROMIS, Prostate cancer

## Abstract

**Background:**

False positive multiparametric magnetic resonance imaging (mpMRI) phenotypes prompt unnecessary biopsies. The Prostate MRI Imaging Study (PROMIS) provides a unique opportunity to explore such phenotypes in biopsy-naïve men with raised prostate-specific antigen (PSA) and suspected cancer.

**Objective:**

To compare mpMRI lesions in men with/without significant cancer on transperineal mapping biopsy (TPM).

**Design, setting, and participants:**

PROMIS participants (*n* = 235) underwent mpMRI followed by a combined biopsy procedure at University College London Hospital, including 5-mm TPM as the reference standard. Patients were divided into four mutually exclusive groups according to TPM findings: (1) no cancer, (2) insignificant cancer, (3) definition 2 significant cancer (Gleason ≥3 + 4 of any length and/or maximum cancer core length ≥4 mm of any grade), and (4) definition 1 significant cancer (Gleason ≥4 + 3 of any length and/or maximum cancer core length ≥6 mm of any grade).

**Outcome measurements and statistical analysis:**

Index and/or additional lesions present in 178 participants were compared between TPM groups in terms of number, conspicuity, volume, location, and radiological characteristics.

**Results and limitations:**

Most lesions were located in the peripheral zone. More men with significant cancer had two or more lesions than those without significant disease (67% vs 37%; *p* <  0.001). In the former group, index lesions were larger (mean volume 0.68 vs 0.50 ml; *p* <  0.001, Wilcoxon test), more conspicuous (Likert 4–5: 79% vs 22%; *p* <  0.001), and diffusion restricted (mean apparent diffusion coefficient [ADC]: 0.73 vs 0.86; *p* <  0.001, Wilcoxon test). In men with Likert 3 index lesions, log_2_PSA density and index lesion ADC were significant predictors of definition 1/2 disease in a logistic regression model (mean cross-validated area under the receiver-operator characteristic curve: 0.77 [95% confidence interval: 0.67–0.87]).

**Conclusions:**

Significant cancer-associated MRI lesions in biopsy-naïve men have clinical-radiological differences, with lesions seen in prostates without significant disease. MRI-calculated PSA density and ADC could predict significant cancer in those with indeterminate MRI phenotypes.

**Patient summary:**

Magnetic resonance imaging (MRI) lesions that mimic prostate cancer but are, in fact, benign prompt unnecessary biopsies in thousands of men with raised prostate-specific antigen. In this study we found that, on closer look, such false positive lesions have different features from cancerous ones. This means that doctors could potentially develop better tools to identify cancer on MRI and spare some patients from unnecessary biopsies.

## Take Home Message

Significant cancer-associated magnetic resonance imaging lesions in biopsy-naïve men with suspected prostate cancer are larger, more conspicuous, and more diffusion restricted than false positives. Prostate-specific antigen density and apparent diffusion coefficient are predictors of significant disease, and could help guide decisions to biopsy men with indeterminate phenotypes.

## 1 Introduction

Although missed significant prostate cancer on multiparametric magnetic resonance imaging (mpMRI) has to be mitigated, the opposite problem, that is, the false positive MRI lesion, obscures the diagnostic process and prompts unnecessary biopsies in biopsy-naïve men with raised prostate-specific antigen (PSA). In PRECISION, the proportion of negative MRI-targeted biopsies was inversely associated with lesion conspicuity (67%, 31%, and 6% for Prostate Imaging Reporting and Data System version 2 [PI-RADS v2] scores of 3, 4, and 5, respectively) and was driven mainly by “indeterminate” or “equivocal” phenotypes, a finding corroborated by the literature [1], [2]. This is an important issue, considering that three in four men with suspected cancer have abnormal mpMRI findings and that the number of those considered for MRI and biopsy every year is set to increase [2], [3], [4].

Unfortunately, although discerning clinically significant prostate cancer (csPCa) from benign processes on mpMRI is crucial, many studies use surgical specimens or inadequately interrogated prostates and are thus limited by selection or biopsy sampling bias [5]. Our aim in this study was to use the unique design of the Prostate MRI Imaging Study (PROMIS) in order to capture the characteristics of false positive MRI lesions and examine how they differ from significant disease-associated phenotypes [6]. This multicentre, paired-cohort, confirmatory study assessed the diagnostic performance of mpMRI against the most stringent reference standard ethically possible. A total of 576 participants underwent mpMRI, followed by combined systematic transrectal ultrasound (TRUS)-guided biopsy and 5 mm transperineal template mapping biopsy (reference test) across the entire prostate, regardless of MRI findings. Owing to the inclusion criteria, blinded design, and use of a stringent reference standard, PROMIS is relatively free of spectrum, verification, and classification biases, despite its limitations.

## 2 Patients and methods

### 2.1 Participants

PROMIS was registered on ClinicalTrials.gov (NCT01292291), and its design has been discussed elsewhere [6]. In brief, this was a multicentre study in which biopsy-naïve men with PSA ≤15 ng/mL underwent prebiopsy 1.5 T mpMRI, followed by a combined biopsy procedure under general anaesthetic. The combined procedure consisted of 5 mm transperineal template mapping (TPM) biopsy followed by standard systematic TRUS-guided biopsy. Each test was performed and reported by clinicians blinded to other results. For this work, only men enrolled at University College London Hospital (UCLH) were considered (*n* = 235).

Ethical approval for PROMIS was granted by the National Research Ethics Service Committee London (Ref: 11/LO/0185).

### 2.2 Study design

Biopsy and imaging data were collected from clinical research reports, including per-patient overall Gleason score and maximum cancer core length (MCCL) on TPM, presenting PSA, prostate volume, and Likert scores, location, volume, and radiological features of individual MRI lesions. Overall Gleason score was defined as the predominant pattern across the entire prostate and constituted the final pathological score. In total, 235 participants were stratified into four mutually exclusive cancer definition groups according to their per-patient TPM summaries (previously recorded by an experienced uropathologist): (1) no cancer, (2) insignificant cancer, (3) secondary/definition 2 significant cancer (Gleason ≥3 + 4 of any length and/or MCCL ≥ 4 mm of any grade), and (4) primary/definition 1 significant cancer (Gleason score ≥4 + 3 of any length and/or MCCL ≥ 6 mm of any grade). Once stratified, men with at least one MRI lesion (Likert score 3–5) were identified for further analyses. For the remainder of this paper, the terms “TPM cancer burden”, “TPM cancer group”, and “TPM group” will be used interchangeably and will refer to TPM grouping according to the four disease definitions described.

### 2.3 Analysis

We summarised baseline characteristics (including presenting PSA, prostate volume, PSA density [PSAD], and MRI lesion counts) using simple statistics such as medians, interquartile ranges (IQRs), and proportions. PSAD was calculated by dividing PSA by the MRI-derived prostate volume (ellipsoid method). We hypothesised that MRI lesions in men with significant disease (definitions 1 and 2) differ from lesions seen in men without significant cancer (no cancer/insignificant cancer) in terms of their prevalence, count, location (peripheral zone [PZ], transition zone, or both), laterality (right, left, or bilateral), focality (focal or diffuse), overall Likert scores, per-sequence Likert scores (T2-weighted imaging [T2WI], diffusion-weighted imaging [DWI], and dynamic contrast-enhanced [DCE] sequences), volume, and apparent diffusion coefficients (ADCs; nonstandardised mean ADCs derived from axial images demonstrating the highest restriction within each lesion). Nonparametric tests (Wilcoxon rank sum and Kruskal-Wallis analysis of variance [ANOVA]) were used to test differences between groups. In men with Likert 3 index lesions, we hypothesised that PSAD and index lesion ADC are predictors of significant disease (definition 1 or 2) in a multivariable binary logistic regression model.

In order to visualise the false positive mpMRI phenotype and further understand its morphology, the prostate borders, transition zone outlines, and any lesions with overall Likert ≥3 in the TPM-negative group were manually contoured in all axial slices of positive mpMRI sequences (ie, individual sequence Likert score ≥3) using the Osirix platform (Pixmeo SARL, Geneva, Switzerland) and the PROMIS pictorial report as a reference. The surfaces of the manually segmented prostate capsule and the transition zones were aligned in a common space using a feature-based, group-wise registration algorithm that iteratively produced a “mean prostate shape” on which lesions can be superimposed, in line with previous work [7]. This algorithm iteratively updates a mean point cloud based on pair-wise alignment between each case and the mean shape until convergence (with apex and base landmarks guiding nonrigid registration). The R statistical software (R Foundation for Statistical Computing, Vienna, Austria; http://www.R-project.org/) was used for all exploratory and statistical analyses, whereas Matlab (MathWorks Inc, Natick, MA, USA) was used for producing lesion density maps. All *p* values were considered significant at the 0.05 level.

## 3 Results

### 3.1 Baseline characteristics

**Fig. 1:**
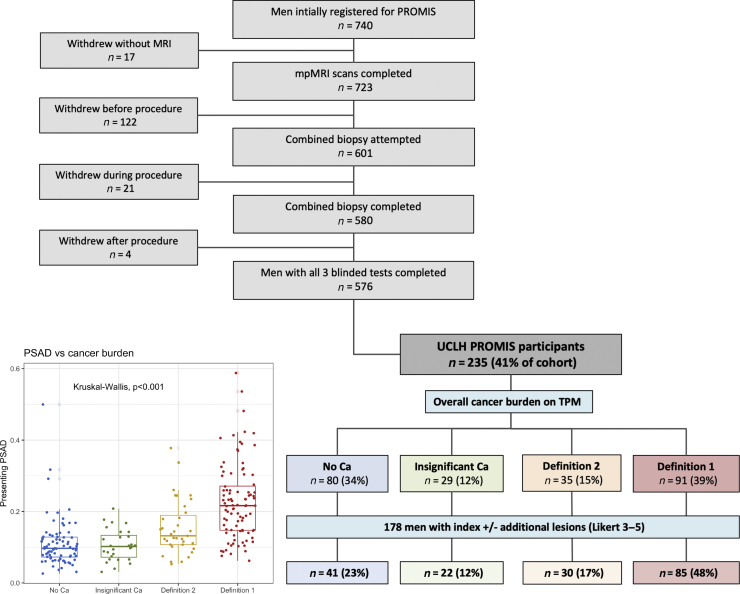
UCLH PROMIS cohort (*n* = 235). Men were classified according to mpMRI findings (Likert scores) and TPM biopsy results to four categories, as shown in the organogram: (1) no cancer, (2) insignificant cancer, (3) definition 1 cancer, and (4) definition 2 cancer. There was a significant difference in presenting PSAD between the four TPM groups (*p* < 0.001, Kruskal-Wallis ANOVA). In total, 178 out of 235 men (76%) had at least one lesion in their prostate and were the focus of subsequent analyses. ANOVA = analysis of variance; Ca = cancer; mpMRI = multiparametric magnetic resonance imaging; PROMIS = Prostate MRI Imaging Study; PSAD = prostate-specific antigen density; TPM = transperineal mapping biopsy; UCLH = University College London Hospital.

The median prostate volume for the entire UCLH cohort (*n* = 235) was 45 ml (IQR: 34–58 ml), and the median presenting PSA value was 6.1 ng/mL (IQR: 4.6–8.5 ng/ml; Supplementary Fig. 1A). The median PSAD (in ng/mL^2^) was significantly different between the four TPM groups (no cancer: 0.10 [IQR: 0.07–0.12], insignificant cancer: 0.10 [IQR: 0.07–0.13], definition 2 cancer: 0.14 [IQR: 0.11–0.20], definition 1 cancer: 0.22 [IQR: 0.15–0.27]; Kruskal-Wallis ANOVA, *p* <  0.001; Fig. 1).

### 3.2 Conspicuity, distribution, and focality of mpMRI lesions (Likert ≥3)

**Fig. 2:**
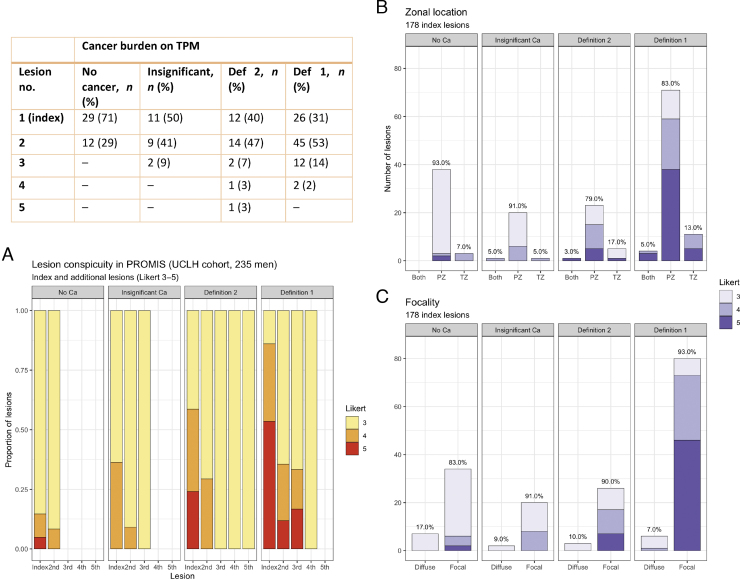
MRI lesion count, conspicuity, distribution, and focality in the UCLH PROMIS cohort. More men with any significant cancer had more than one lesion (77/115 or 67%) than those without significant disease (23/63 or 37%, *p* <  0.001; see the table). (A) The proportion of index lesions with high conspicuity (Likert score 4–5) increased with significant cancer burden and was significantly higher in men with significant cancer (91/115, 79%) than in those without significant disease (14/63, 22%; *p* <  0.001). (B) Index lesions were predominantly distributed in the PZ across all groups. (C) MRI lesions characterised by the uroradiologist as “diffuse” were more common in the TPM-negative/insignificant disease groups collectively than in men with significant disease, although this difference was not statistically significant. Ca = cancer; MRI = magnetic resonance imaging; PROMIS = Prostate MRI Imaging Study; PZ = peripheral zone; TPM= transperineal mapping biopsy; TZ = transition zone.

In total, 178 out of 235 (76%) men had at least one lesion in their prostate (Fig. 1). The proportion of men with at least one lesion was higher in those with definition 1/2 csPCa (115/126, 91%) compared with those without/insignificant cancer (63/109, 58%; *p* <  0.001, chi-square test for proportions). More men with csPCa had two or more lesions (77/115, 67%) than those without/insignificant cancer (23/63, 37%), and the percentage difference was statistically significant (*p* <  0.001). Furthermore, lesion conspicuity was associated with disease significance (Fig. 2A): in men with csPCa (both definitions), 91/115 (79%) index lesions were scored as Likert 4–5 versus 14/63 (22%) in men without/insignificant cancer (*p* <  0.001). Similar trends were observed for secondary and tertiary lesions (Fig. 2A).

Index lesions were predominantly located in the PZ in 93%, 91%, 80%, and 85% of men without cancer, with insignificant disease, with definition 2 disease, and with definition 1 disease, respectively, and the differences were not significant (Fig. 2B). In total, 14% of index lesions were reported as “diffuse” in men without csPCa versus 8% in those with significant cancer (*p* =  0.2), and the largest difference in the proportion of diffuse lesions was between the TPM-negative and definition 1 disease groups (17% vs 7%, *p* =  0.08; Fig. 2C).

### 3.3 Radiological characteristics of mpMRI lesions (Likert ≥3)

**Fig. 3:**
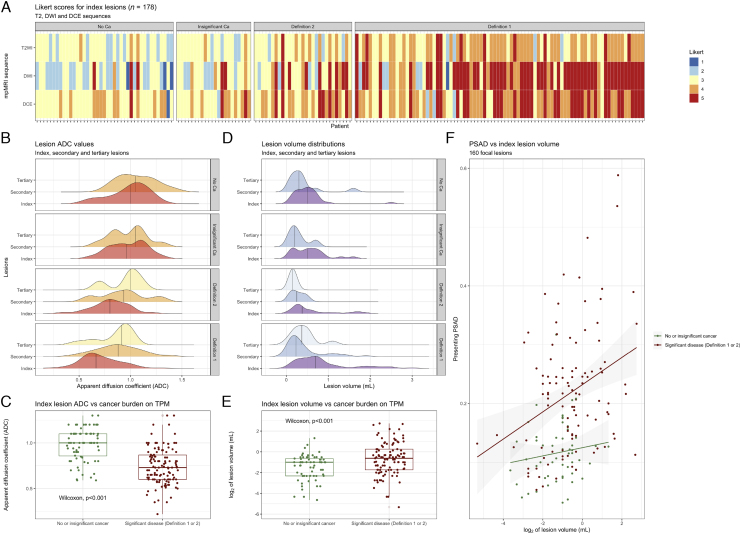
Basic radiomic characteristics of mpMRI lesions across TPM groups in the UCLH PROMIS cohort. (A) As the burden of significant disease on TPM increased, so did Likert scores for all mpMRI sequences; however, there was also a gradual shift from predominantly T2W + DWI–DCE + MRI phenotypes in men without significant cancer to T2W++DWI+++DCE++ lesions in those with significant disease. (B and C) Lesion ADC values decreased with increasing disease burden (ADC distributions shown; two outliers removed for visualisation purposes), but this reduction was particularly marked for index lesions. (D and E) Lesion volumes were greater and more skewed in men with definition 1 disease (volume distributions shown; seven outliers removed for visualisation purposes), particularly in the case of index lesions. (F) There was a positive relationship between lesion volume and PSAD, especially in men with significant cancer (regression lines shown for men with/without significant disease). ADC = apparent diffusion coefficient; Ca = cancer; DCE = dynamic contrast enhanced; DWI = diffusion-weighted imaging; mpMRI = multiparametric MRI; MRI = magnetic resonance imaging; PROMIS = Prostate MRI Imaging Study; PSAD = prostate-specific antigen density; TPM = transperineal mapping biopsy; T2W = T2 weighted.

More lesions were positive in all three mpMRI sequences in men with significant disease than in those without (97/115 [84%] vs 30/63 [48%], *p* <  0.001). Per-sequence Likert scores of index lesions were overall higher in men with significant disease, but concurrently, there was a gradual change from a T2W+DWI–DCE+ to a T2W+DWI+DCE+ phenotype with increasing cancer burden (Fig. 3A). The proportion of T2WI-positive lesions was higher in men with significant disease than in those without (107/115 [93%] vs 48/63 [76%], *p* =  0.002), as was the proportion of DWI-positive lesions (105/115 [91%] vs 36/63 [57%], *p* <  0.001). This was not true for the proportion of DCE-positive lesions (112/115 [97%] vs 61/63 [97%]), although the degree of DCE positivity increased in csPCa groups (Fig. 3A). The DWI Likert score inversion in prostates with significant cancer was corroborated by the ADC distributions (Fig. 3B), confirming significant index lesion ADC differences between men with and those without csPCa (*p* <  0.001, Wilcoxon test; Fig. 3C). A similar ADC trend was observed for secondary and tertiary MRI lesions, although less marked (Fig. 3B).

**Fig. 4:**
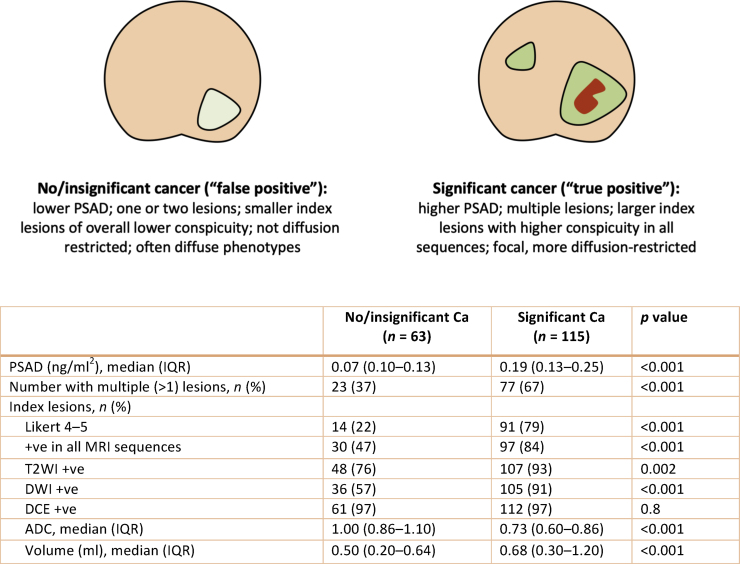
Summary of the main clinical-radiological differences between men with and without significant cancer. False and true positives differ in terms of their overall PSAD level, presence or absence of additional MRI lesions, and features of index lesions (such as overall conspicuity, diffusion restriction, and volume). ADC = apparent diffusion coefficient; Ca = cancer; DCE = dynamic contrast enhanced; DWI = diffusion-weighted imaging; IQR = interquartile range; MRI = magnetic resonance imaging; PSAD = prostate-specific antigen density; T2WI = T2-weighted imaging.

Most index lesions were smaller than 1 ml, but men with significant disease had skewed volume distributions (Fig. 3D) and higher index lesion volumes compared with those without csPCa (*p* <  0.001, Wilcoxon test; Fig. 3E). Similarly, the median PSAD was higher in men with “true positive” lesions than in those with “false positives” (0.19 vs 0.07, *p* <  0.001, Wilcoxon test), and there was a positive relationship between lesion volume and PSAD, particularly in the csPCa group (Fig. 3F). A summary of all the clinical-radiological differences between “false” and “true positives” (which could be useful for their discrimination) is given in Fig. 4. More refined ADC and volume comparisons between all four TPM groups are presented in Supplementary Fig. 2.

### 3.4 PSAD and ADC as predictors of significant cancer in men with Likert 3 index lesions

**Fig. 5:**
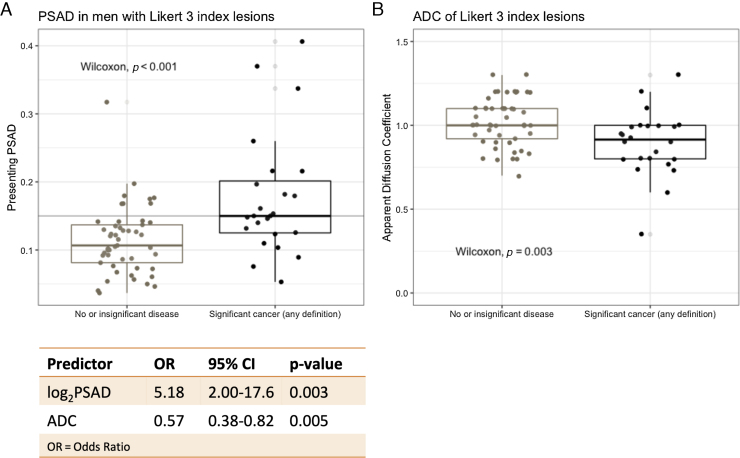
Detection of significant cancer in men with Likert 3 index lesions. There was a significant difference in (A) PSAD and (B) index lesion ADC values between men with significant cancer and those without (PSAD threshold of 0.015 shown in grey). Both log2PSAD and index lesion ADC were significant predictors of definition 1 or 2 cancer in a binary logistic regression model (refer to the table; odds ratios shown for every PSAD doubling and for every 0.1 [ie, 100 × 10^–6^ mm^2^/s] increase in ADC). The 10-fold cross-validated mean AUC for the model was 0.77 (95% CI: 0.67–0.87). ADC = apparent diffusion coefficient; AUC = area under the receiver-operator characteristic curve; CI = confidence interval; OR = odds ratio; PSAD = prostate-specific antigen density.

Since indeterminate phenotypes are the main drivers of MRI-positive/biopsy-negative discrepancies in men with suspected cancer, we tested the ability of PSAD and ADC to predict significant cancer in men with Likert 3 index lesions (*n* = 73). Of these men, 49 (67%) had no/insignificant cancer, whereas the remaining 24 (33%) had significant cancer of any definition. There were statistically significant PSAD and index lesion ADC differences between men with csPCa and those without (*p* <  0.001, Wilcoxon test; Fig. 5A and 5B). More refined PSAD and ADC comparisons between all four TPM groups in men with Likert 3 lesions are presented in Supplementary Fig. 3.

The ability of PSAD and index lesion ADC to predict significant disease was evaluated through binary logistic regression, where the positive outcome was definition 1/2 cancer on TPM and the negative outcome was no/insignificant cancer. The area under the receiver operating characteristic curve (AUC) for either log_2_PSAD or ADC alone was 0.75 (95% confidence interval [CI]: 0.63–87.7) and 0.72 (95% CI: 0.59–0.85), respectively, whereas, in a combined multivariable model, both log_2_PSAD (*p* =  0.003) and index lesion ADC (*p* =  0.005) were significant predictors of significant cancer (mean 10-fold cross-validated AUC: 0.77 [95% CI: 0.67–0.87]; refer to the table in Fig. 5). The full model’s net benefit was overall higher than that of a “biopsy all men” approach: decision curve analysis showed that at a 10% risk threshold (ie, assuming that nine unnecessary biopsies per detected significant cancer is a reasonable cost:benefit ratio), 325 men could be spared from biopsy for every 1000 significant cancers detected (Supplementary Fig. 4).

### 3.5 Multiparametric MRI lesions in TPM-negative men

**Fig. 6:**
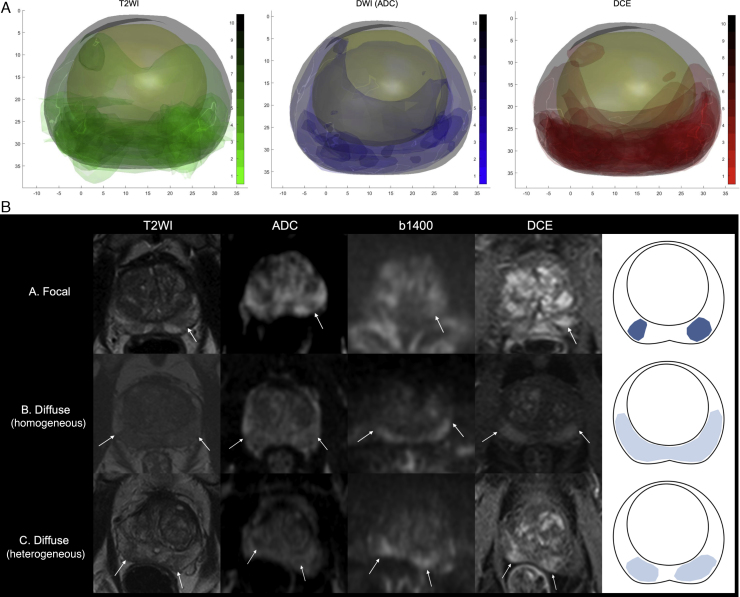
False positive mpMRI phenotypes in TPM-negative men (*n* = 77; three excluded due to incomplete DICOM data). Prostate outlines, transition zones, and all Likert 3–5 lesions (41 index and 12 additional) were annotated on the Osirix platform (T2W, ADC, and DCE sequences; all axial slices). (A) Lesions in each positive sequence are superimposed on a “mean prostate shape” for the TPM-negative group, as described in the Methods. The produced maps confirm a PZ distribution and dominance of a T2W + DWI–DCE + phenotype. (B) Index lesions could broadly be divided into three morphological categories: focal (*n* = 34), diffuse homogeneous (*n* = 4), and diffuse heterogeneous (*n* = 3). Typical examples of lesions of each category are shown in T2W, ADC, b1400, and DCE sequences (white arrows), along with a diagrammatic representation of the three types. ADC = apparent diffusion coefficient; DCE = dynamic contrast enhanced; DWI = diffusion-weighted imaging; mpMRI = multiparametric magnetic resonance imaging; PZ = peripheral zone; TPM = transperineal mapping biopsy; T2WI = T2-weighted imaging.

T2, ADC, and DCE prostate and lesion density maps were constructed for 77 TPM-negative men, as described in the Methods. Forty-one men had at least one MRI lesion and 12 had two (index and one secondary). The maps confirmed that lesions were predominantlydistributed in the PZ and had a T2W + DWI–DCE +  phenotype (Fig. 6A). Morphologically, MRI index lesions in this subgroup could broadly be divided into different types: 34 focal (83%) and seven diffuse (17%), with four diffuse homogeneous and three diffuse but heterogeneous (Fig. 6B). The vast majority of index lesions were scored as Likert 3 (including all diffuse ones), with only six focal lesions scored as Likert 4 or 5 (Fig. 2C). All 12 secondary lesions were focal with a score of Likert 3, apart from one scored as Likert 4 in a man with a Likert 5 index lesion.

## 4 Discussion

We investigated MRI lesions in biopsy-naïve prostates thoroughly interrogated regardless of prior imaging findings. We focused on false positive MRI phenotypes, which are often attributed to insignificant cancer and conditions such as benign hyperplasia and inflammation [8]. Although per-prostate Likert scores were available, we classified MRI-positive men based on their index or additional lesions (designated as such by the reporting uroradiologist), allowing a more refined analysis of the MRI phenotypes seen in the biopsy-naïve prostate. We found that both “false positive” and significant cancer-associated lesions were located predominantly in the PZ, with corroborating studies suggesting that a false positive reading cannot be reliably associated with zonal location [9]. However, men with significant disease had multiple, larger, and more conspicuous lesions, with a concomitant shift to more diffusion-restricted phenotypes, as evidenced by their disproportionately higher DWI Likert scores and lower ADC distribution ranges.

We produced preliminary evidence that simple, readily available MRI-derived metrics such as PSAD and ADC can predict csPCa in biopsy-naïve men with indeterminate (Likert 3) index lesions and TPM as a reference. We did not externally validate our model and the size of our sample was relatively small, but internal cross-validation resulted in consistently high performance. Although we would anticipate some loss of performance in real-life MRI-targeted settings, the potential of PSAD to predict significant disease has been demonstrated in men with a prior biopsy, and studies correlating imaging with prostatectomy specimens or TRUS biopsy tissue confirm that radiomic features (including ADC) can differentiate tumours from benign processes, particularly in the PZ [10], [11], [12], [13], [14], [15]. Of note, standardisation of ADC values against normal prostate or urine on diffusion imaging by an experienced uroradiologist did not significantly change either the main results or model performance (Supplementary Fig. 5). Finally, we visualised the spatial distribution of MRI lesions in men without any cancer using group-wise registration across all three mpMRI sequences, which is a useful and novel addition to the literature.

Our analyses are post hoc and based on data from a single institution, thus limiting the immediate clinical application of our findings. In addition, we relied on a single overall TPM pathological score based on Gleason/MCCL instead of a refined zonal assessment of significant disease, as the clinicians performing TPM were blinded to MRI results and individual lesions were not targeted. Nonetheless, overall TPM pathological scores were assigned by an experienced uropathologist, and per-patient analysis mirrors real-life diagnostic settings. Furthermore, computer simulations and studies correlating 5-mm mapping with surgical specimens have shown that TPM, which is the most stringent reference standard that can be applied in a biopsy-naïve population, reflects the true disease state within a given prostate [16], [17], [18]. Although many centres use a version of the PI-RADS system for assessing MRI lesions, Likert score was used in the PROMIS study, the design of which predated existing PI-RADS versions [19], [20]. Likert is currently recommended by the UK National Institute for Health and Care Excellence, and the diagnostic agreement between the two systems has been demonstrated previously [10], [21], [22], [23], [24].

## 5 Conclusions

Although most MRI lesions in biopsy-naïve men with suspected cancer are located in the PZ, phenotypes associated with clinically significant disease tend to be more conspicuous and diffusion-restricted. Metrics such as MRI-calculated PSAD and ADC could be clinically useful predictors of significant disease in men with indeterminate phenotypes. Further research will focus on the independent validation of these findings.

  ***Author contributions*:** Vasilis Stavrinides had full access to all the data in the study and takes responsibility for the integrity of the data and the accuracy of the data analysis.

  *Study concept and design*: Stavrinides, Ahmed, Emberton.

*Acquisition of data*: Syer, Giganti, Freeman, Bott, Brown, Frangou, Burns-Cox, Dudderidge, Bosaily, Ghei, Henderson, Hindley, Kaplan, Oldroyd, Parker, Persad, Rosario, Shergill, Carmona Echeverria, Norris, Winkler, Punwani, Kirkham.

*Analysis and interpretation of data*: Stavrinides, Hu.

*Drafting of the manuscript*: Stavrinides, Whitaker, Emberton.

*Critical revision of the manuscript for important intellectual content*: Syer, Giganti, Freeman, Bott, Brown, Frangou, Burns-Cox, Dudderidge, Bosaily, Ghei, Henderson, Hindley, Kaplan, Oldroyd, Parker, Persad, Rosario, Shergill, Carmona Echeverria, Norris, Winkler, Punwani, Kirkham, Barratt.

*Statistical analysis*: Stavrinides, Karapanagiotis.

*Obtaining funding*: Stavrinides, Ahmed, Emberton.

*Administrative, technical, or material support*: Barratt, Hu.

*Supervision*: Whitaker, Emberton.

*Other*: None.

  ***Financial disclosures:*** Vasilis Stavrinides certifies that all conflicts of interest, including specific financial interests and relationships and affiliations relevant to the subject matter or materials discussed in the manuscript (eg, employment/affiliation, grants or funding, consultancies, honoraria, stock ownership or options, expert testimony, royalties, or patents filed, received, or pending), are the following: Freeman and Kirkham have shares in Nuada Medical Ltd. Hindley has stock or share interest with Nuada, is Clinical Director for the Prostate Care Division, and has also received funding from Sonacare for teaching and training. Norris receives research funding from the MRC. Carmona Echeverria receives funding from Prostate Cancer UK. Punwani has sessional funding from UCLH BRC. Ahmed's research is supported by core funding from the UK's National Institute of Health Research (NIHR) Imperial Biomedical Research Centre. Ahmed currently receives funding from the Wellcome Trust, Prostate Cancer UK, The Urology Foundation, BMA Foundation, Imperial Healthcare Charity, Sonacare Inc., Trod Medical, and Sophiris Biocorp for trials in prostate cancer. Travel allowance was previously provided from Sonacare. Ahmed is a paid medical consultant for Sophiris Biocorp and Sonacare Inc, and is a proctor for Boston Scientific for Rezum and cryotherapy. Emberton receives funding from NIHR-i4i, MRC, Sonacare Inc., Trod Medical, Cancer Vaccine Institute, and Sophiris Biocorp for trials in prostate cancer. Emberton is a medical consultant to Sonacare Inc., Sophiris Biocorp, Steba Biotech, GSK, Exact Imaging, and Profound Medical, and has stock interest in Nuada Medical Ltd. Travel allowance was previously provided from Sanofi Aventis, Astellas, GSK, and Sonacare. Ahmed and Emberton are proctors for HIFU with Sonacare Inc. and paid for training other surgeons in this procedure. Hu and Barratt are former shareholders in SmartTarget Ltd., a UK-based UCL spin-out company founded to commercialise image fusion software for assisting targeted prostate cancer biopsy and focal therapy. The other authors declare no competing interests.

  ***Funding/Support and role of the sponsor*:** PROMIS was funded by the UK Government Department of Health, National Institute of Health Research–Health Technology Assessment Programme (Project no.: 09/22/67) and by the UCLH/UCL Biomedical Research Centre and the Royal Marsden and Institute for Cancer Research Biomedical Research Centre. The analysis presented here was funded by the Medical Research Council through a Clinical Research Training Fellowship awarded to Vasilis Stavrinides (Ref: MR/S005897/1). Vasilis Stavrinides is supported by an MRC Clinical Research Training Fellowship (MR/S005897/1) and acknowledges previous support from EACR (EACR Travel Fellowship) and UCL (Bogue Fellowship). Vasilis Stavrinides and Solon Karapanagiotis are supported by The Alan Turing Institute under the EPSRC grant EP/N510129/1. Francesco Giganti is funded by a UCL Graduate Research Scholarship and the Brahm PhD Scholarship in memory of Chris Adams. Alex Kirkham is supported by the UCLH/UCL Biomedical Research Centre. Joseph Norris acknowledges research funding from the MRC (MR/S00680X/1). Mark Emberton is a United Kingdom National Institute of Health Research (NIHR) Senior Investigator and receives research support from the UCLH/UCL NIHR Biomedical Research Centre.
