# Supplementary material for: False Positive Multiparametric Magnetic Resonance Imaging Phenotypes in the Biopsy-naïve Prostate: Are They Distinct from Significant Cancer-associated Lesions? Lessons from PROMIS[image]
Source: Eur Urol. 2021 Jan;79(1):20–9. doi: 10.1016/j.eururo.2020.09.043 (PMC7772750; doi:10.1016/j.eururo.2020.09.043)
Supplement: Supplementary file 1 [file mmc1.docx]

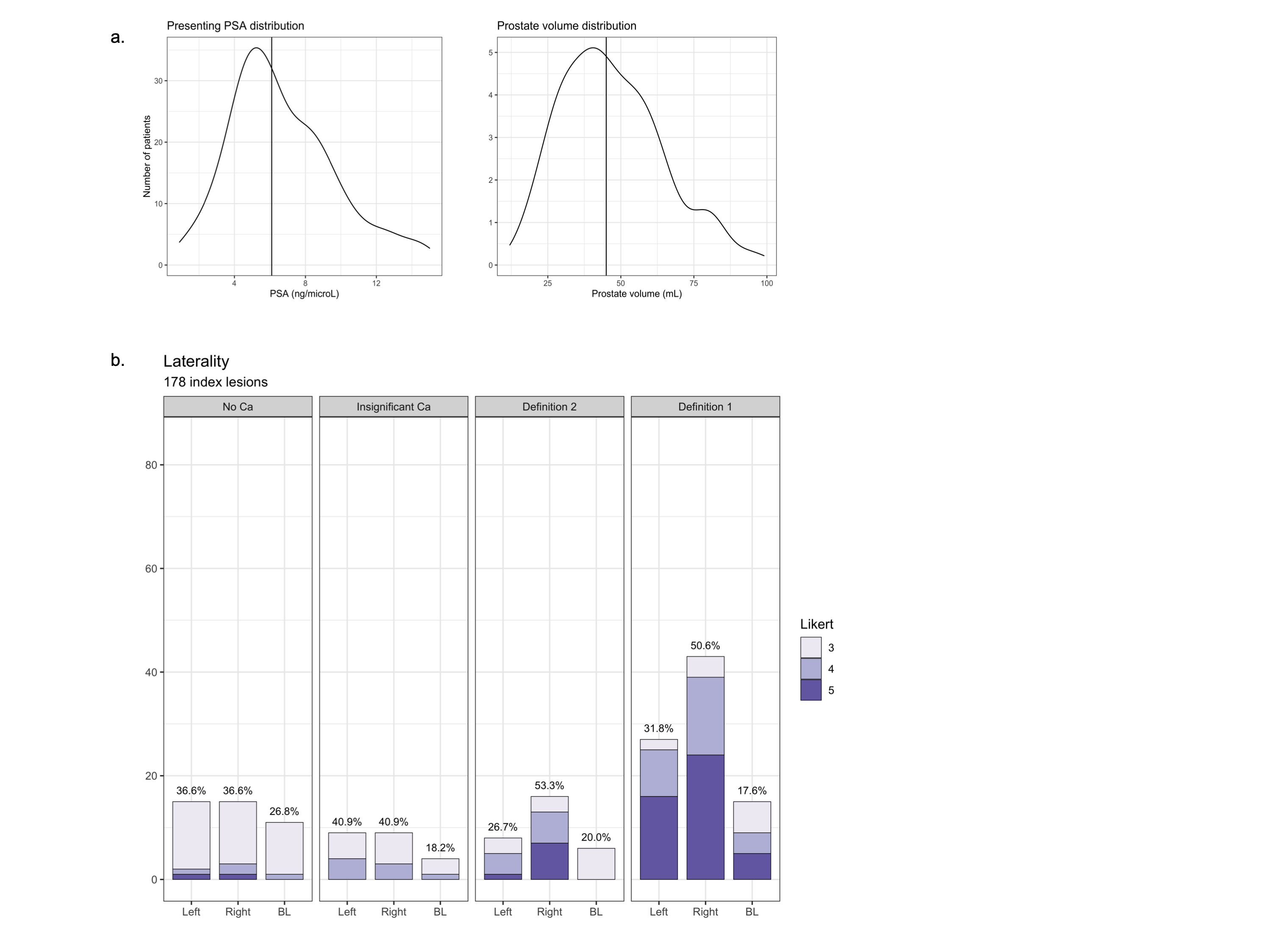


**Supplementary Figure 1.** (a) PSA and prostate volume distributions for the entire UCLH PROMIS cohort (medians shown as black vertical lines). (b) Index lesion laterality. There were no statistically significant differences in the proportion of lesions distributed to the left or right side of the prostate in the no/insignificant cancer groups. However, in those with significant cancer there was a preference for the right side compared to the left (53% vs 27% in Definition 2; 51% vs 32% in Definition 1).


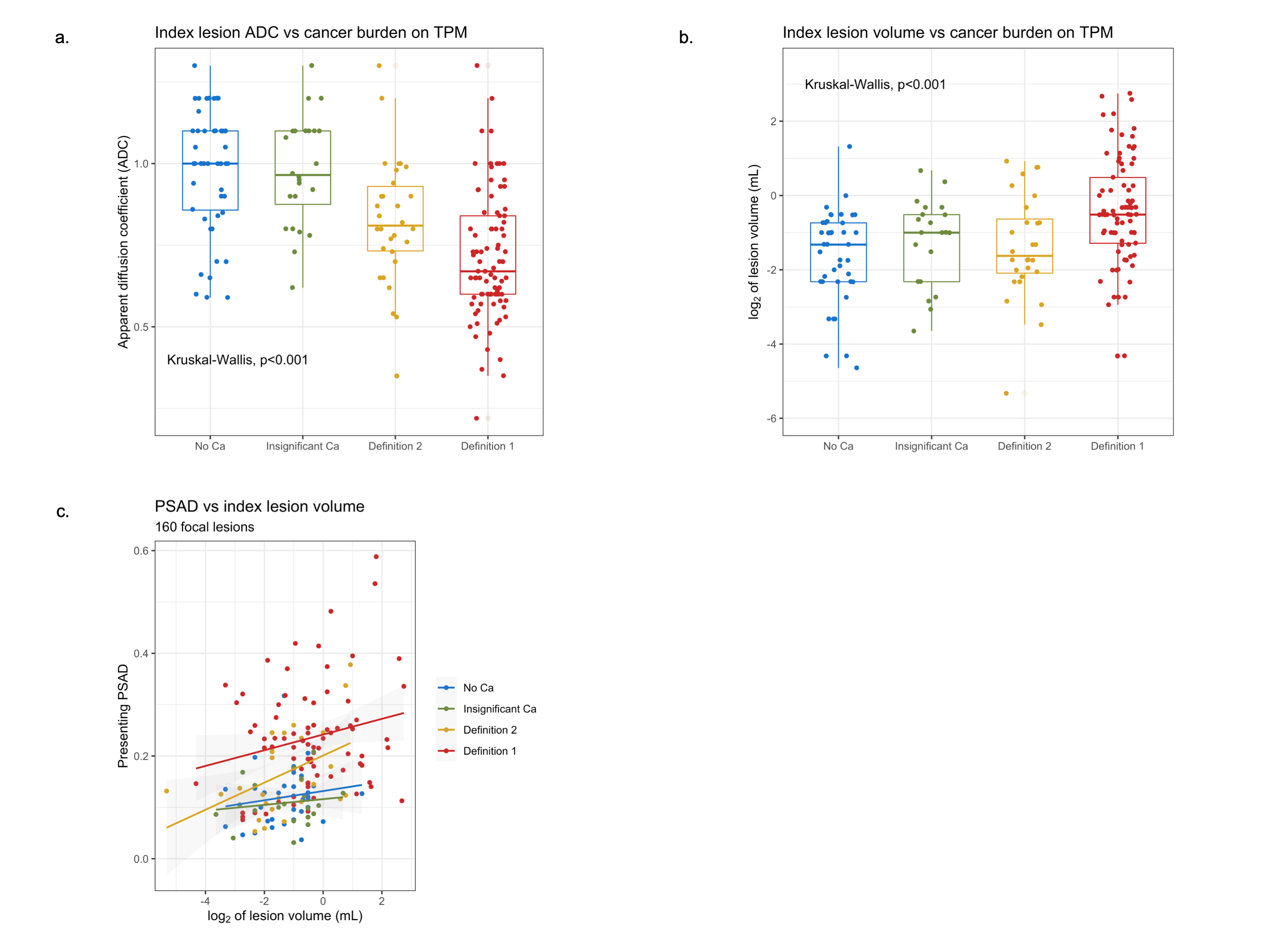


**Supplementary Figure 2.** Index lesion ADC (a) and volume (b) differences in shown between all four TPM groups. Also, the relationship between PSAD and lesion volume is shown for all groups (c).


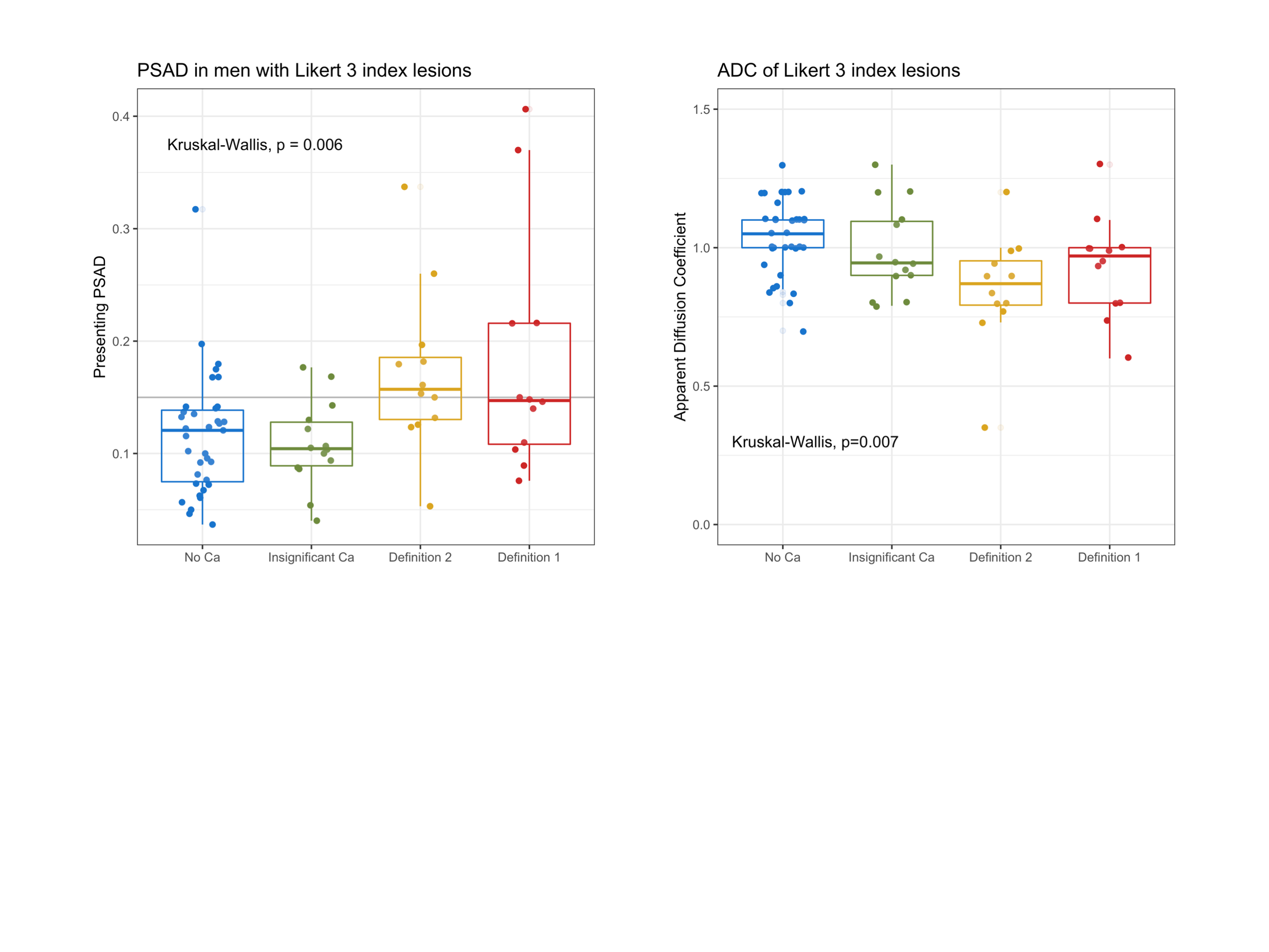


**Supplementary Figure 3.** PSAD and index lesion ADC differences across all four TPM groups in men with Likert 3 index lesions (n=73).


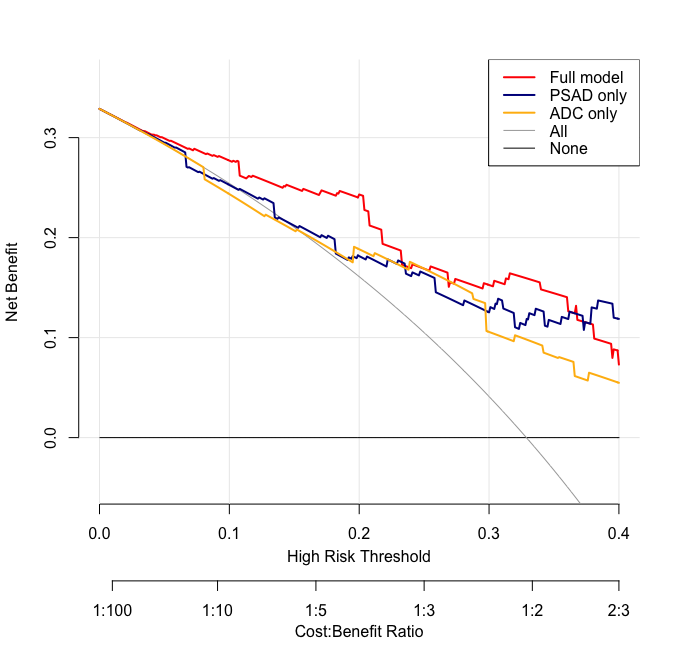


**Supplementary Figure 4.** Evaluation of the logistic regression model predicting significant disease in men with Likert 3 index lesions. The AUCs for either log_2_PSAD or index lesion ADC as predictors were 0.75 (95 % CI: 0.63-87.7) and 0.72 (95% CI: 0.59-0.85) respectively, whereas for the combined model the 10-fold cross validated mean AUC was 0.77 (95% CI: 0.67-0.87). Net benefit curves are shown for the full model (red), models using either predictor alone (PSAD only in blue; index ADC only in gold) and a “biopsy all” approach (grey), indicating that the combined model’s net benefit is higher for most relevant risk thresholds compared to other approaches. Assuming a risk threshold of 10%, the net benefit difference of the combined model versus a “biopsy all” approach was 0.3253, indicating that 325 men would be spared from biopsy for every 1000 significant cancers detected if the model were to be applied. These findings have to be interpreted carefully due to the small size of the Likert 3 subgroup, the lack of independent validation and the use of TPM as a reference standard.

|  | Prostate-normalized ADC | Urine (bladder)-normalized ADC |
| --- | --- | --- |
| ADC  distributions | 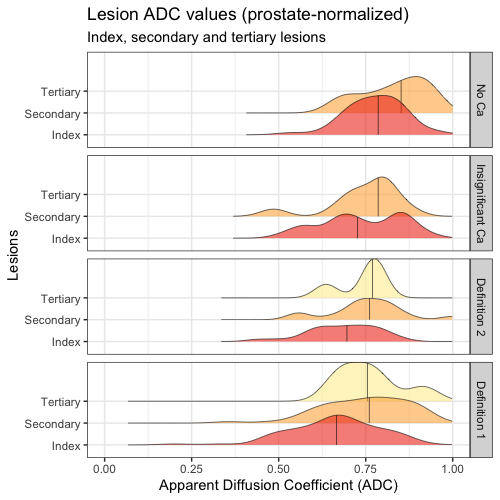 | 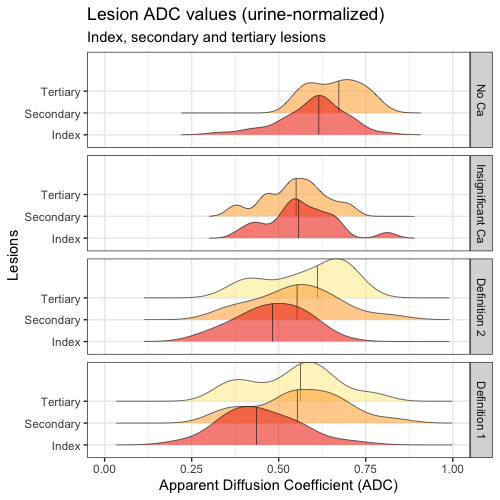 |
| ADC comparisons (Def1/2 cancer vs no/insignificant) | 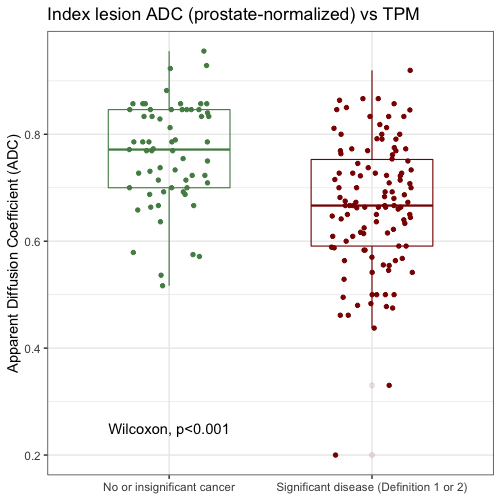 | 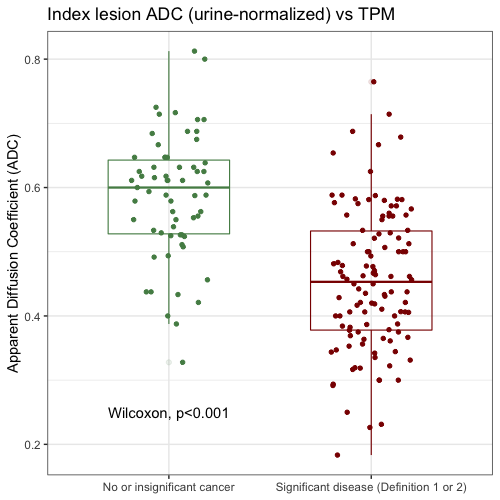 |
| ADC comparisons (Def1/2 cancer vs no/insignificant) -  Likert 3 index lesions | 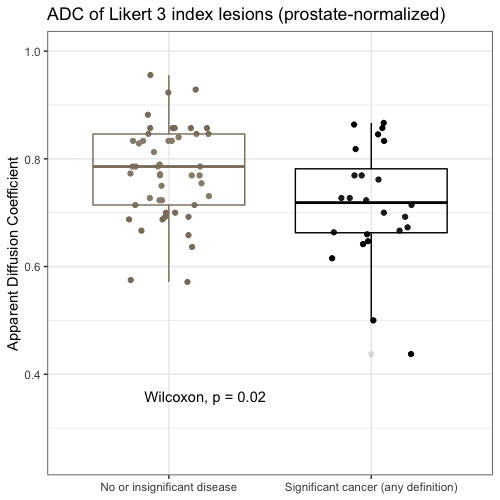 | 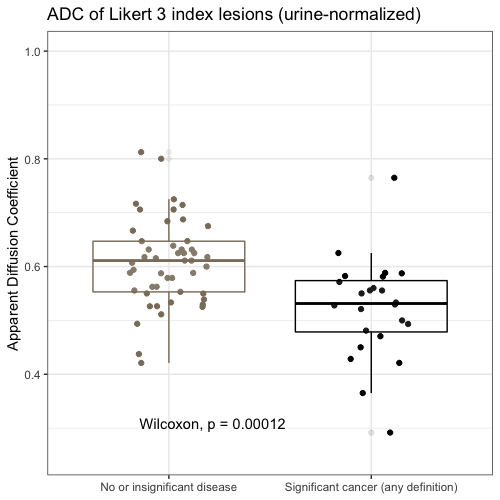 |
| Logistic regression model | 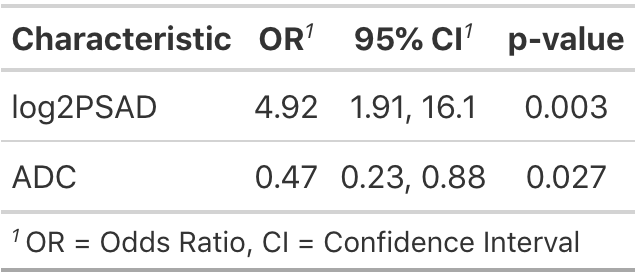 | 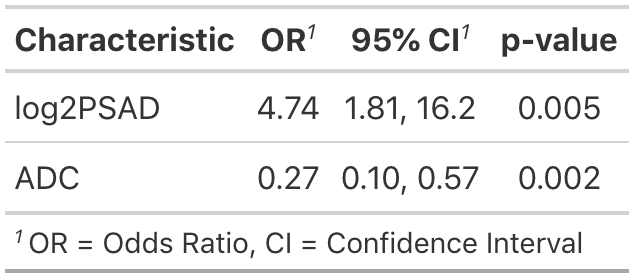 |
| Model mean CV AUC | 0.76 (95% CI: 0.66-0.87) | 0.79 (95% CI: 0.65-0.94) |

**Supplementary Figure 5.** Analyses based on normalized ADC values. An experienced uro-radiologist (FG; 7 years of experience in prostate mpMRI; reporting more than 1,500 prostate MR scans per year) reviewed the images of all men with lesions Likert 3-5 and obtained ADC values for the urine/bladder as well as from the normal prostate in mirror position to each lesion (as described in Giganti et al. *Magnetic Resonance Imaging* 2020; 67:50-58. doi: 10.1016/j.mri.2019.12.007). ADC normalization did not alter the main conclusions of the study, regardless of the fraction used (ADC_lesion_/ADC_normal prostate_ or ADC_lesion_/ADC_urine_).
